# Stability of Ar/O_2_ Plasma-Treated Polypropylene Membranes Applied for Membrane Distillation

**DOI:** 10.3390/membranes11070531

**Published:** 2021-07-14

**Authors:** Marek Gryta, Wirginia Tomczak

**Affiliations:** Faculty of Chemical Technology and Engineering, West Pomeranian University of Technology in Szczecin, ul. Pułaskiego 10, 70-322 Szczecin, Poland

**Keywords:** hydrophobic membrane, membrane distillation, plasma treatment, surface hydrophilization

## Abstract

In the present work, Ar/O_2_ plasma treatment was used as a surface modification tool for polypropylene (PP) membranes. The effect of the plasma conditions on the properties of the modified PP surface has been investigated. For this purpose, the influence of gas composition and its flow rate, plasma power excitation as well as treatment time on the contact angle of PP membranes has been investigated. The properties of used membranes were determined after various periods of time: immediately after the modification process as well as after one, four and five years of storage. Moreover, the used membranes were evaluated in terms of their performance in long-term MD process. Through detailed studies, we demonstrated that the performed plasma treatment process effectively enhanced the performance of the modified membranes. In addition, it was shown that the surface modification did not affect the degradation of the membrane matrix. Indeed, the used membranes maintained stable process properties throughout the studied period.

## 1. Introduction

Properties and surface structure of the membrane are the key parameters influencing separation effects in membrane processes. Indeed, the membrane pore size determines the size of the retained particles and macromolecular substances in microfiltration (MF) and ultrafiltration (UF) processes, respectively, while the porous support dense skin layer enables separation of ions from desalinated water by the reverse osmosis process (RO) [1,2,3].

It has been widely documented, e.g., [4,5,6,7], that water desalination can also be performed by the membrane distillation process (MD). However, it is necessary to mention that since in MD, process water and other volatile compounds evaporate from a feed through pores filled by gas phase, the solutes rejection is influenced by the vapour–liquid equilibrium. For this reason, in order to separate the feed from the distillate obtained, it is necessary to maintain a vapor layer inside the membrane. Most often, it is obtained using porous membranes made of highly hydrophobic polymers such as polypropylene (PP) [8], polytetrafluoroethylene (PTFE) [9] and polyvinylidenefluoride (PVDF) [10].

It should be noted that fouling occurring during membrane exploitation is a critical issue and has serious consequences for the process efficiency and performance. Hence, preventing a decline in permeate flux is a basic requirement for membrane process application on an industrial scale [11,12]. As has been indicated by Yao et al. [4], key factors influencing fouling and wetting in the MD process are classified as: membrane surface properties, process parameters and feed characteristics. An increasing number of studies have found that reducing of the fouling intensity can be achieved by various methods of modifying the surface properties of the membranes [13,14,15,16,17,18,19]. For example, it has been demonstrated [20,21] that fouling caused by hydrophobic compounds present in the feed can be successfully limited by improving the membrane’s surface hydrophilicity. Surface deposition, interfacial crosslinking, coating and graft polymerization are applied for surface modification [22,23,24,25].

A relatively simple, fast and effective method of membrane modification is plasma treatment, which has also been successfully used in the modification of membranes for MD applications [26,27,28,29,30,31,32]. The process of plasma treatment allows one to induce hydrophilicity of the polymer surface [33]. Undoubtedly, the advantage of the plasma treatment process is the fact that it does not change the properties of the bulk of the membrane matrix, but only modifies its surface, usually several nanometers deep [34]. Various methods and design solutions of plasma induction apparatuses are used, which significantly affects the obtained surface modification effects [25]. In addition, for a given apparatus, various effects can be obtained by changing the composition of plasma gases as well as its operating parameters, such as time of plasma treatment and excitation power. This may hinder the process of membrane modification, as it requires very careful selection of parameters for its implementation [35]. On the other hand, in an industrial application, using only one apparatus and making slight changes to the parameters ensure producing membranes with different performances [25].

Summarizing the literature data, it can be concluded that since the plasma gas composition determines the effectiveness of the process, the selection of suitable gases is a key issue. When using inert gases such as argon (Ar) or helium (He), the obtained plasma gas does not contain new components that can be deposited on the membrane surface, as is the case with, e.g., CF_4_ plasma [30,31]. However, Ar or He plasma may cause etching/degradation of the polymer surface which changes surface morphology and porosity [36]. Gas plasma, which contains air, O_2_, CO_2_ or H_2_O demonstrates a similar effect [35,37]. In turn, the presence of oxygen in the plasma promotes the formation of hydrophylic groups [38]. Moreover, free radicals excited during the action of the plasma are still active and cause further changes in the composition of the surface. As a result, if the material is in contact with air, oxidation processes may occur and hydrophilic groups such as COOH or C=O are formed on the surface, although the plasma did not contain oxygen [39].

In the case of hydrophobic polymers such as polypropylene, it is advantageous to hydrophilize their surface, which reduces organic fouling and increases adhesion to, e.g., paints. Unfortunately, it was found that the created hydrophilic surface quickly regains its hydrophobicity. This is due to the fact that some hydrophilic compounds (low molecular oxidized compounds—LMOC) evaporate from the membrane surface or, as a result of reorganization of polymer chains, penetrate into its deeper layers [33,35]. In the case of the MD process, it can be expected that the recovery of hydrophobicity will be advantageous since higher hydrophobicity leads to less surface wetting. However, some hydrophilic groups should remain on the membrane surface to limit the deposition of hydrophobic foulants [26]. 

When assessing the properties of the membranes, the time from the production of the membranes to the start-up of the membrane system should also be taken into account. Indeed, this is a period of various multi-stage activities and certainly lasts at least several months. Therefore, it is important to investigate the changes in membrane properties, e.g., one year after their modification. In addition, the plasma treatment process may initiate polymer degradation during the storage of modules or reveal it during the operation of the MD installation.

Taking the abovementioned into account, the objective of this study was to investigate stability of Ar/O_2_ plasma-treated PP membranes applied for membrane distillation. The properties of used membranes were determined immediately after the modification process as well as after one, three, four and five years of storage.

## 2. Materials and Methods

### 2.1. Plasma Treatment

The capillary polypropylene membranes (Table 1) manufactured for the microfiltration process by Microdyne GmbH (Germany) were applied for the presented studies. The membranes have a sponge structure with an average pore diameter of 0.2 μm. 

The membranes were modified by gas plasma using an apparatus for plasma enhanced chemical vapor deposition (PECVD) 2.45 GHz produced by Roth & Rau (Wüstenbrand, Germany). In order to obtain the plasma, Ar or a mixture of Ar/O_2_ of the following concentration (volume%): Ar80%–O_2_20% or Ar50%–O_2_50% was used. Analytical grade Ar and O_2_ gases were used. The applied gas flow rate was in the range of 30–90 mL/min. The PECVD system used microwave excitation at a frequency of 2.45 GHz. The plasma power was in the range of 50–205 W. Additionally, the sample holder was connected to the direct current (DC) voltage, which by applying negative DC bias voltages (Bias) allows the increase of the energies of the ions bombarding the surface of the treated sample [40]. In our studies, the values of Bias were varied from 80 to 203 V. 

Four membrane samples, spaced 5 mm apart, were placed in the chamber of the apparatus on the substrate holder. The chamber was vacuumed, and then plasma was generated at 6–7 Pa for a defined time. Plasma duration time equal to 1, 3 and 5 min was used. The plasma stream, acting from the top, modified only half of the external surface of the capillary membrane. Therefore, after plasma treatment was completed, the membranes were inverted and the process was repeated. The results of the studies using a scanning microscope showed that such treatment did not create the boundary between the lower and upper surfaces of the membranes. The obtained modified membrane samples were stored in the closed plastic containers at ambient conditions. 

### 2.2. Membrane Distillation

**Figure 1:**
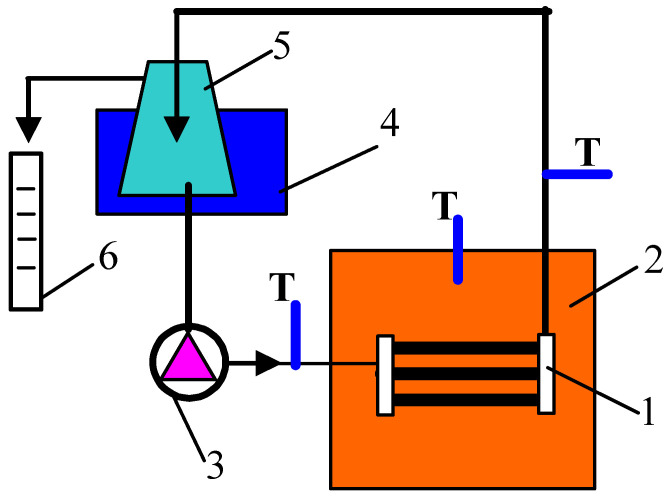
Experimental Membrane Distillation (MD) set-up. 1—MD module, 2—feed tank with electrical heating, 3—peristaltic pump, 4—cooling bath, 5—distillate tank, 6—measurement cylinder, T—thermometer.

The MD process was performed in the direct contact MD (DCMD) configuration using modules without external housing (submerged module) with distillate flow inside the PP capillaries. Such a system has been successfully applied to treatment of oily wastewaters and construction of membrane bioreactors [41,42]. The MD experimental studies were carried out using the installation schematically shown in Figure 1. Three capillary membranes were mounted in each submerged MD module with an effective length of 8 cm (module area of 19.6 cm^2^). The tested MD module was placed in a feed tank that was electrically heated. An industrial temperature controller EDIG (Nűga Company, Germany) was used. The applied feed temperature was 353 ± 0.5 K. The volume of the feed in the tank was kept constant, V_F_ = 4.3 ± 0.1 L. In part of the presented study, a laboratory thermostat (5 L) was used as the feed tank. Inside the membranes, the cooled distillate (300 ± 2 K) flowed at a speed of 0.35 m/s. The MD process was carried out continuously by collecting the permeate flowing from the distillate loop in the distillate tank. The permeate flux (L/m^2^h) was calculated as the mean of the distillate volume collected over 24 h. NaCl solutions and salt solution contaminated by oil were used as a feed. 

### 2.3. Analytical Methods

The composition of the membrane surface was tested using the attenuated total reflection. Attenuated Total Reflection-Fourier Transform Infrared Spectroscopy (ATR-FTIR) analyses were performed using a Nicolet 380 FTIR spectrophotometer coupled with Smart Orbit diamond ATR accessory (Thermo Electron Corp., Waltham, MA, USA).

Scanning Electron Microscopy (SEM) (Hitachi SU8000, Tokyo, Japan) and an Atomic Force Microscopy (AFM) (multi-mode 8 AFM, Brucker, Santa Barbara, CA, USA) were used to study the membrane morphology.

The membrane hydrophobicity was determined by water contact angle (WCA). The sessile drop method using the Contact Angle System OCA (Data Physics, Filderstadt, Germany) apparatus was used for the WCA measurements. The measurements were performed for each type of studied membranes, repeated five times across the surface of the sample and the mean value of WCA was determined. 

Diameters of membrane pores were measured via a mercury porosimetry technique using Autopore III (Micrometrics GmbH, Aachen, Germany). 

Distillate conductivity was measured with a 6P Ultrameter (Myron L Company, Carlsbad, CA, USA). 

The oil content in the solutions was examined by infrared method using an oil analyzer OCMA 500 manufactured by Horiba (Kyoto, Japan).

## 3. Results and Discussion

### 3.1. Conditions of Plasma Treatment

It is well established that plasma treatment is a complex process and its conditions may affect the properties of the polymer surface [43]. Therefore, in the first stage of the presented studies, the effect of the plasma treatment conditions on the properties of the modified PP surface has been investigated. For this purpose, various values of the gas flow velocity and different gas compositions were used. Moreover, a wide range of the plasma operating time and excitation power was applied. The obtained effects were assessed by measuring the water contact angle. In order to avoid the possible influence of the porosity of the capillary wall, a nonporous flat PP foil (0.2 mm thickness) was used for preliminary tests.

**Figure 2:**
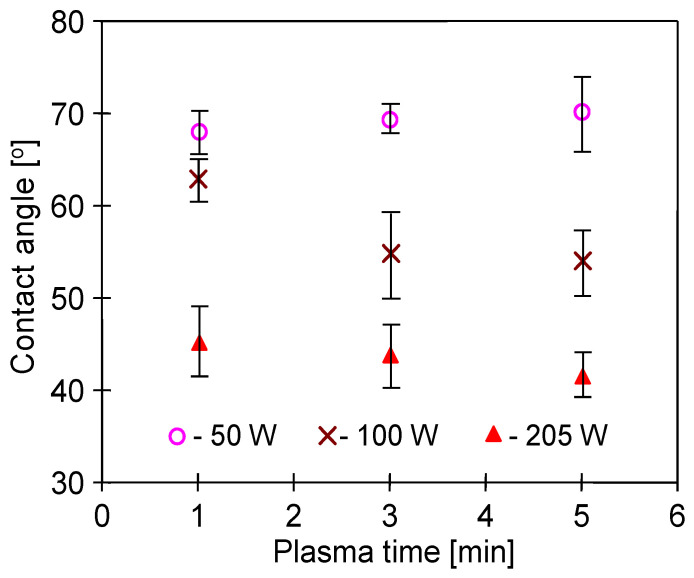
Effect of Ar plasma excitation power on the contact angle of polypropylene (PP) foil. Ar flow rate: 90 mL/min.

**Figure 3:**
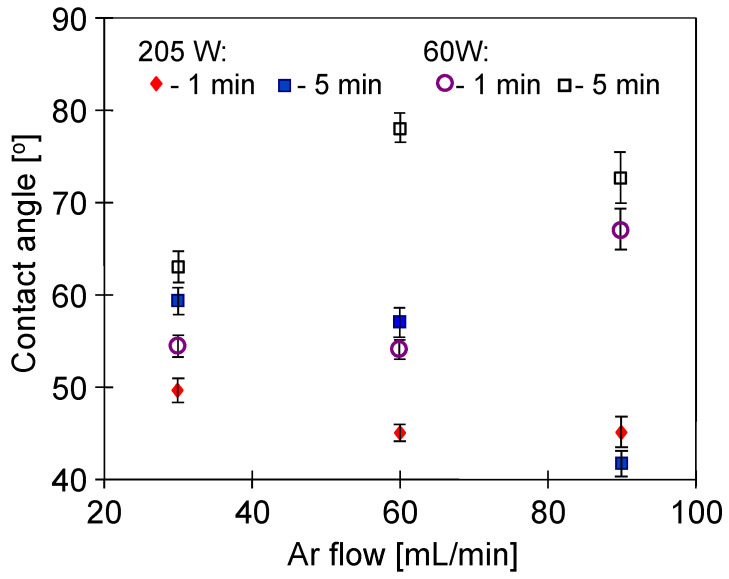
Ar plasma. Effect of gas flow rate, plasma treatment time and power on the contact angle of PP foil.

Contact angle of the non-treated PP foil was equal to 95° and, as a result of the plasma treatment, it decreased significantly (Figure 2). It has been determined that each of the tested parameters had a significant impact on the changes in the hydrophobicity of the PP foil surface. These results are in good agreement with the work of [35], where similar changes in contact angle (below 70°) were obtained for polypropylene membranes modified by Ar plasma. As has been indicated by the authors, the use of low power caused the hydrophobicity to quickly increase to over 80°. In general, lower and more stable contact angle values are obtained by increasing the power of the plasma excitation [44]. This observation was confirmed in the presented studies, regardless of both plasma exposure time (Figure 2) and gas flow (Figure 3). Increasing the gas flow promotes the homogeneity of the produced plasma [26]. On the other hand, the increase in plasma power results in greater intensity of free radicals activity, leading to an increase in the content of oxygen compounds on the membrane surface [45]. In addition, changing the surface roughness also affects the CA value. Indeed, the plasma etches the polymer surface and at the same time the concentration of the polymer in the gas phase increases, which results in its redeposition on the sample surface, leading to an increase in the surface roughness. Importantly, even small changes in plasma treatment parameters may significantly affect the etching/redeposition balance [46], resulting in different changes in CA values (Figure 3).

**Figure 4:**
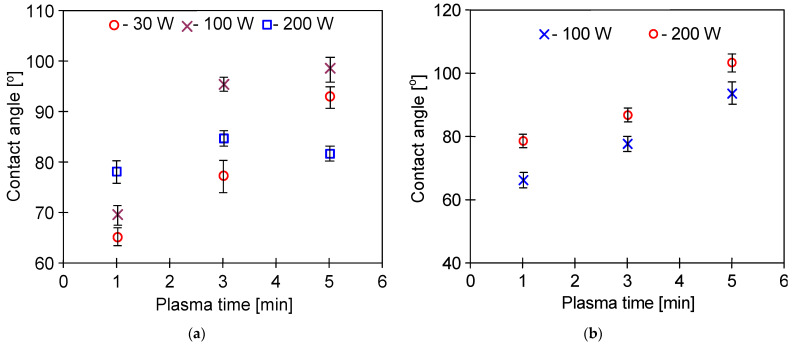
Effect of Ar/O_2_ plasma power on the contact angle of PP foil: (**a**) gas plasma: Ar 80% and 20% O_2_. Flow rate: 90 mL/min; (**b**) gas plasma: Ar 50% and 50% O_2_, flow rate: 90 mL/min.

**Figure 5:**
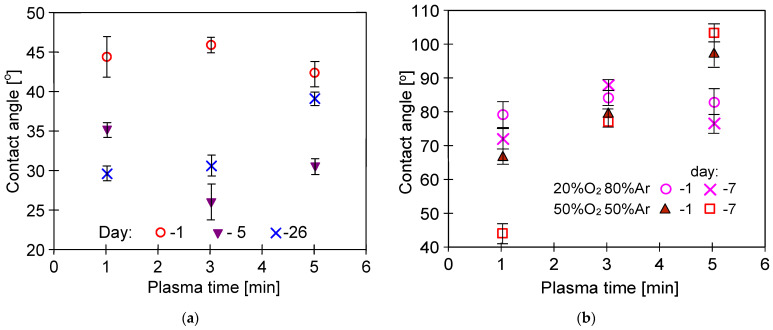
Durability of plasma effects: (**a**) plasma: pure Ar (1–26 days); (**b**) plasma: Ar–oxygen mixture (1–7 days). Plasma power 200 W and flow rate: 90 mL/min.

A much smaller reduction in contact angle was noted when oxygen was added to Ar (Figure 4 and Figure 5). This effect was observed despite the fact that the presence of oxygen in the plasma gas generally increases the number of oxygen-based polar groups, which in turn increases the hydrophilicity of the surface [37]. However, in another study [47], it has been shown that initially, the plasma causes etching of the surface, while the extension of its time (4 and more minutes) favors the redeposition of fragments on the polymer surface, which leads to increasing its hydrophobicity.

Notably, several researchers observed the phenomenon of the rapid recovery of membrane hydrophobicity within 1–2 days after plasma exposure [36,45]. In this study, good stability of the obtained changes was reported, especially for higher values of the plasma excitation power (Figure 5). Moreover, due to a plasma treatment on the PP surface, low molecular oxidized compounds (LMOCs) were created. The hydrophilic LMOCs are weakly bound to the polymer surface and can be easily removed from its surface by rinsing with water or disappear during the membranes storage (ageing phenomenon) [36]. This confirms the conclusion presented by Wade et al. [48], indicating that the gas effect depends on the polymer type. 

### 3.2. Parameters of Membrane Modification

The studies carried out with the use of PP foil showed that application of higher plasma power did not destroy the polymer. Moreover, good stability of the CA value was obtained, which confirms the results of the other study [45]. For these reasons, high power values (100–205 W) were used for modifying the PP membranes. The contact angle values presented in Table 2 indicate that the surface of the modified membranes had hydrophilic properties. Contrary to PP foils, also for the Ar/O_2_ plasma, a significant reduction of the contact angle value was obtained. This may limit the intensity of the fouling phenomenon caused by hydrophobic substances, but, on the other hand, such low values (55–65°) can result in wetting of surface pores during the MD process.

**Figure 6:**
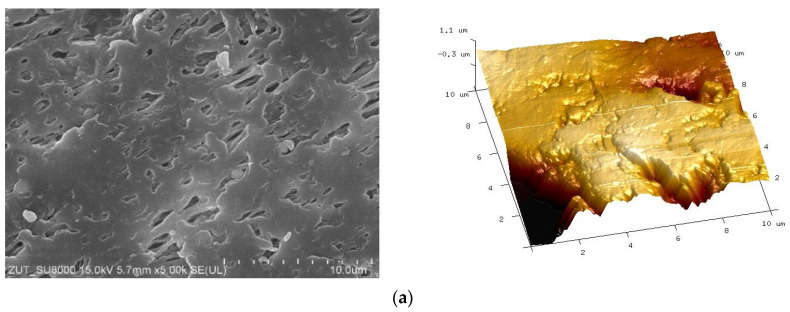

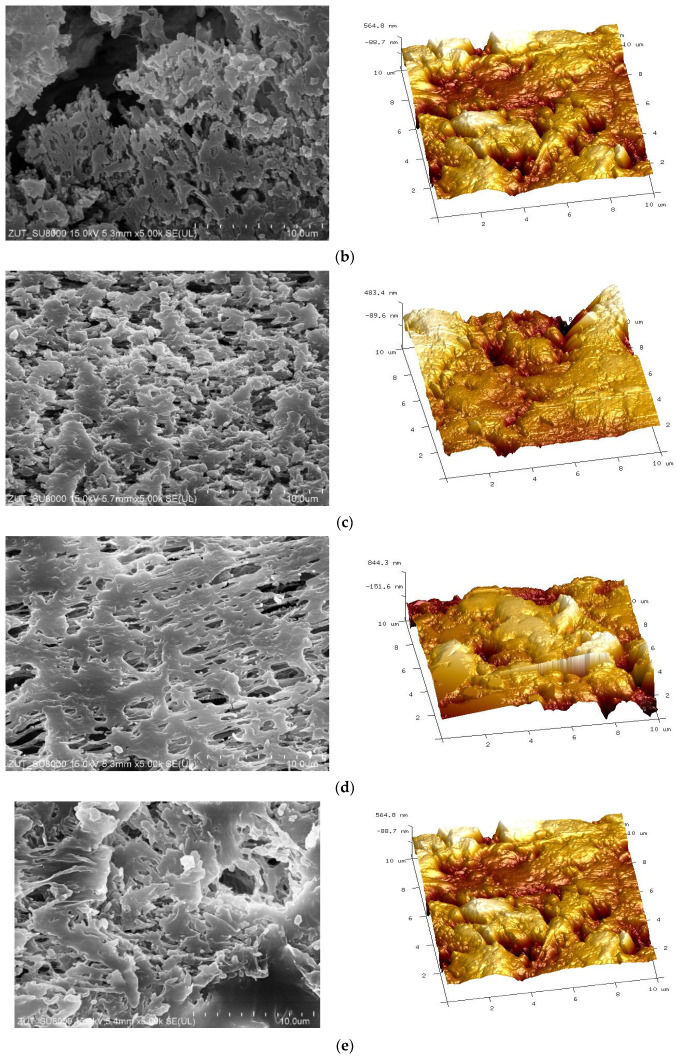

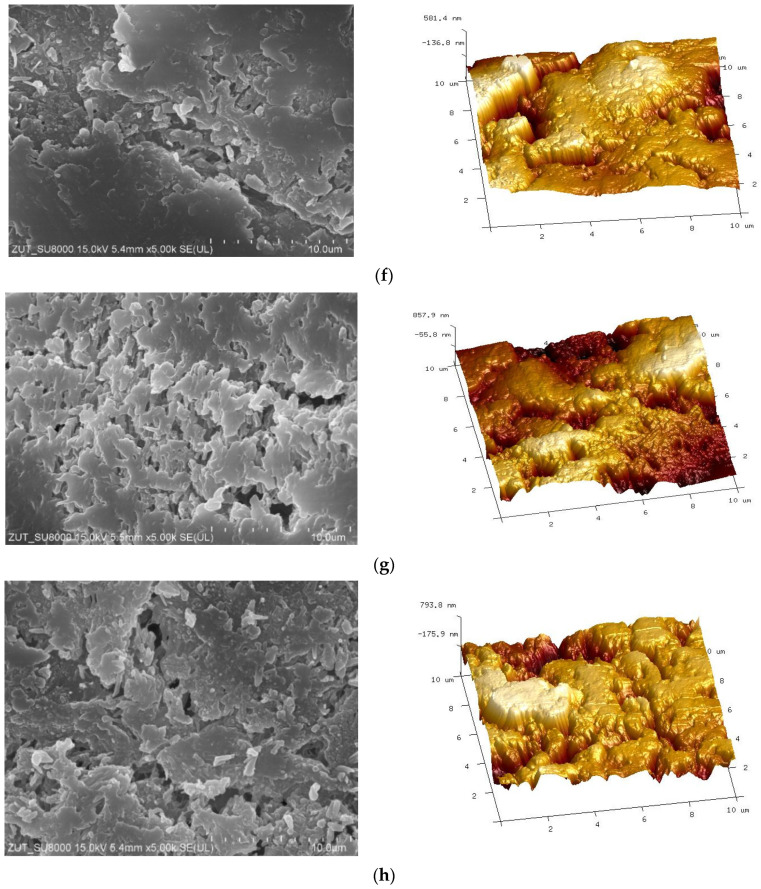
Scanning Electron Microscopy (SEM) (the left side) and Atomic Force Microscopy (AFM) (the right side) surface images of studied membranes: (**a**) #0; (**b**) #1; (**c**) #2; (**d**) #3; (**e**) #4; (**f**) #5; (**g**) #6; (**h**) #7.

**Figure 7:**
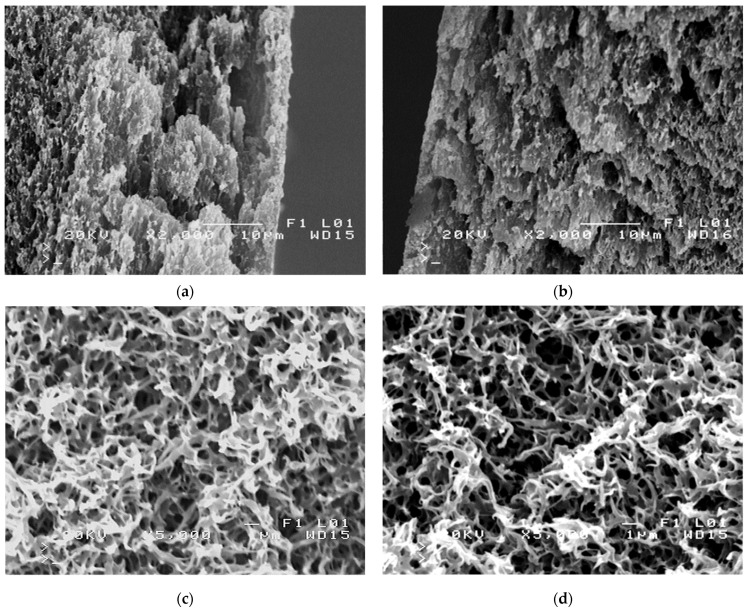
SEM images of the membrane cross-section. Virgin membrane #0: (**a**) edge of external surface, (**c**) inside the wall. Membrane #6: (**b**) edge of external surface, (**d**) inside the wall.

The SEM and AFM studies (Figure 6) showed that the plasma treatment process significantly modified the membrane surface. The obtained results confirm that even small changes in the plasma formation parameters resulted in significant differences in the morphology of the treated membrane surface. Higher R_A_ values were reported for Ar plasma-modified membranes. In study [49], it has been shown that though different gases exhibit different etching rates of polymers, Ar is more powerful in roughening the surface through physical etching under strong ion bombardment. However, it has been found that the plasma treatment process led to modification only of the membrane surfaces. Indeed, the SEM studies of the membrane cross-section (Figure 7) did not demonstrate significant changes in the pore membranes’ walls, which indicates that they maintained their properties. 

**Figure 8:**
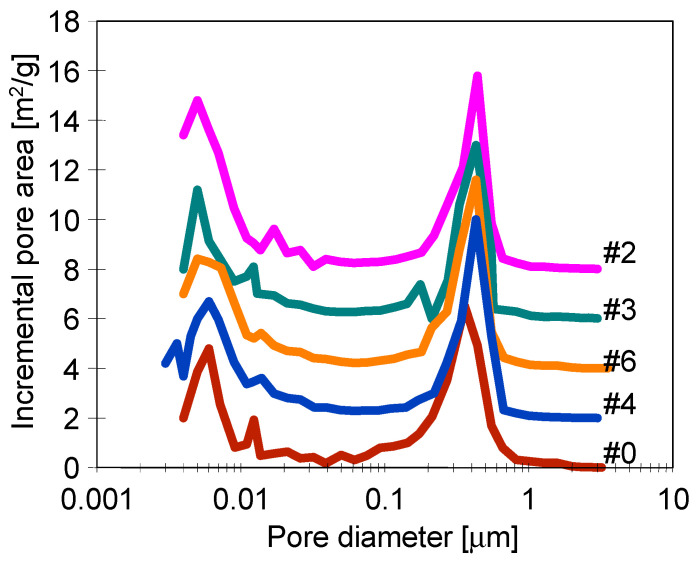
Pore size distribution determined by a mercury porosimetry method.

It is important to note that the results obtained from the studies performed by a mercury porosimetry method (Figure 8) confirmed that the observed changes in pore size were related only to the membrane surface, while the interior of the pore wall did not change. Compared to membrane #0, results for the membranes treated by plasma showed a slight shift of the peak in the range of 0.4–0.6 µm, indicating the greater proportion of large pores formed on their surface. As a result of the use of plasma, the larger pores were formed only on the membrane surface, which is advantageous for MD membranes since the larger pores are faster wetted by the feed. Moreover, it is expected that the formation of hydrophilic compounds will facilitate surface wetting. However, as shown in the previous work [26], wetting the thin surface layer with plasma did not cause a significant decrease in the MD process efficiency. This is due to the fact that the water conductivity coefficient is higher than that of the dry membrane; therefore, filling of the surface pores by water does not reduce the temperature of the evaporation surface.

### 3.3. Membrane Wettability

**Figure 9:**
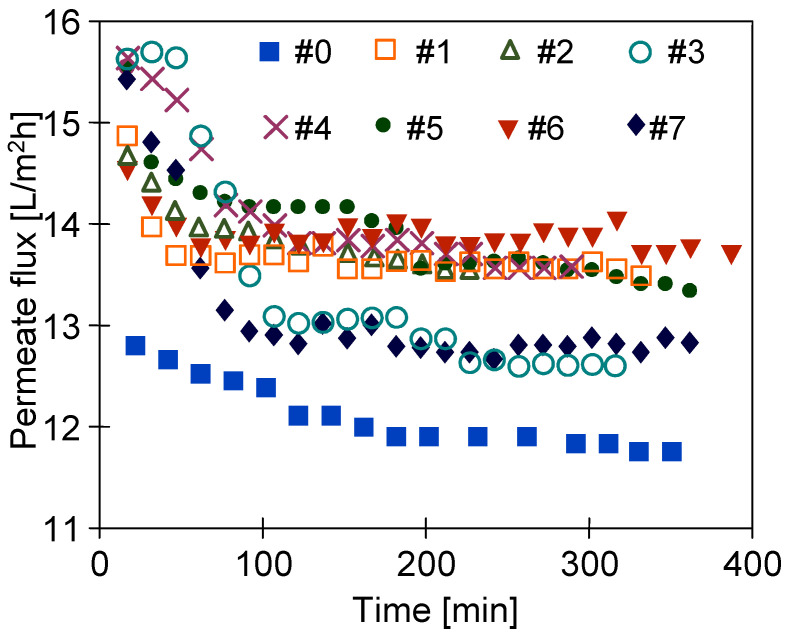
Changes in permeate flux during the MD process with membranes treated by plasma. Feed: NaCl solution (5 g/L). One month after plasma treatment.

Figure 9 shows the changes in permeate flux during the MD process of 5 g NaCl/L solution with membranes treated by plasma. It can be seen that the performed surface modification allowed a significant increase in the efficiency of the process. During the first 2 h of the processes, the permeate flux decreased and, finally, stabilized at the level of 0.85–0.93 of the maximum value. This could be due to the wetting of the surface pores by the feed. Generally, the surface layer by water filling does not significantly affect the MD process conditions [26]. However, in the case of NaCl solution, evaporation of it inside the pores causes a significant increase in the salt concentration at the feed/vapor interface. According to Roult’s law, this causes a reduction in the vapor pressure, i.e., a reduction in the driving force for mass transport in the MD process. The diaphragm layer (laminar sublayer) is stationary; there is no feed flow inside the wetted pores. In this case, only diffusion (dC/dx) may provide the salt back transport from the wetted pores to the feed bulk.

Wetting the surface pores can facilitate the wetting of the entire membrane wall, which should significantly increase the distillate conductivity. However, it was shown that the conductivity of the distillate was less than 5 µS/cm (feed conductivity over 10,000 µS/cm). This observation confirmed that the used plasma hydrophically modified only the membrane surface. In turn, inside the membrane wall, the pores were still hydrophobic, which prevented them from being wetted. 

**Figure 10:**
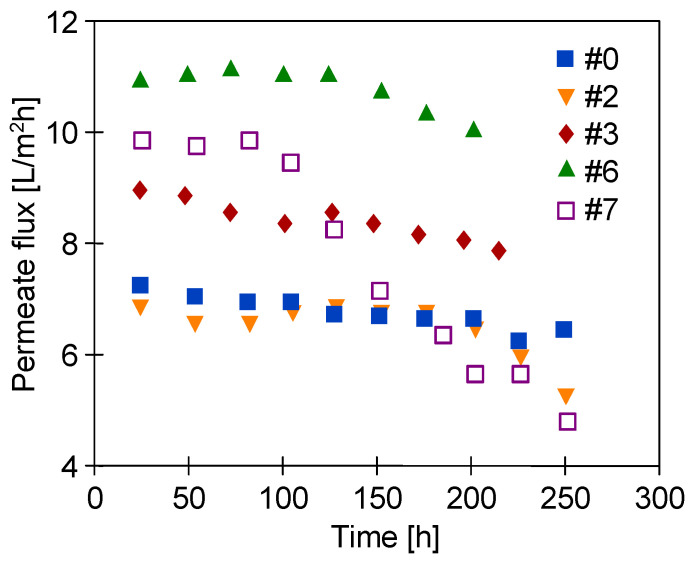
Changes in permeate flux during long-term water desalination by the MD process. One year after plasma treatment.

After the experiments were completed, the MD modules were rinsed thoroughly with distilled water and, after drying, they were placed in a plastic box filled with air for one year. After this period, the membranes were still flexible and MD processes were resumed. For study, the following membranes were selected: membranes characterized by the similar high performance (membranes #2 (Ar) and #6 (Ar/O_2_)) and the lowest performance (membranes #3 (Ar) and #7 (Ar/O_2_)) during the initial studies (Figure 9). The experiments were conducted continuously for 250 h (Figure 10). For safety reasons, the laboratory thermostat (Figure 1, element 2) was replaced by a heating chamber with an industrial controller. Since no feed mixing was performed, the noted permeate flux (Figure 10) was slightly lower than those shown in Figure 9. This is due to the fact that turbulence of the feed flow has a significant impact on the membrane surface temperature. Indeed, no mixing led to a decrease in the temperature value (lower value of the heat transfer coefficient), which in turn reduced the value of the driving force of mass transport in MD [26].

**Figure 11:**
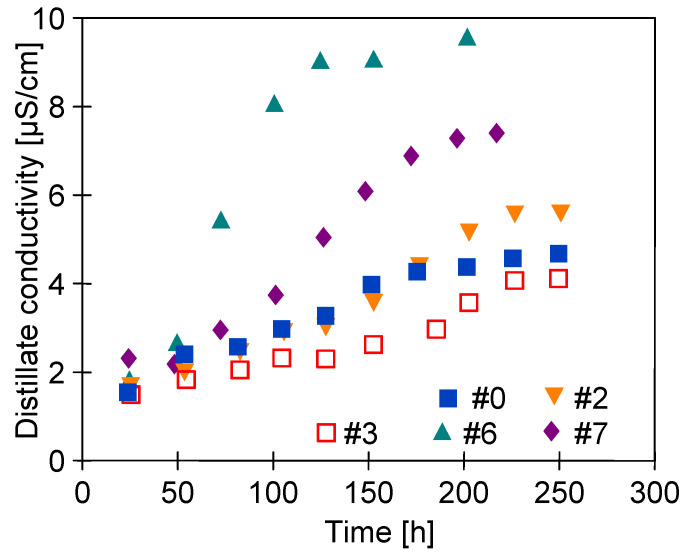
Changes in distillate electrical conductivity during the MD process (Figure 10). One year after plasma treatment.

It must be recognized that despite the long service life of the membranes (250 h), the membranes demonstrated a stable performance. Moreover, the distillate conductivity only increased slightly (Figure 11). This observation confirms the high resistance of the membranes to wetting. Remarkably, the exception has been noted for the #7 membrane, for which the performance after 100 h of the process rapidly decreased to the value equal to 50% of the maximum permeate flux. However, the conductivity of the distillate did not exceed 4 μS/cm (Figure 11, #7). Therefore, it can be concluded that the change in #7 membrane performance was not caused by wetting of the pores. Moreover, the performance obtained for membranes #2 and #6 (similar at the beginning of the study, Figure 9) now differed significantly. In both cases, it was due to changes in the porosity of the surface layer (see the next section).

### 3.4. Long-Term Stabilization of Membranes

After 4 years from the plasma treatment process, both the unused membranes and those in the MD modules, when tested, were still characterized by good flexibility. Although no changes in the value of the contact angle have been reported for the #0 membrane, significant changes for membranes treated by plasma have been observed (Table 3). For instance, the contact angle for the #2 membrane treated by Ar plasma after 4 years of storage increased from 62.8 ± 3.5° to 106.2 ± 3.4°. In turn, for the #6 membrane treated by Ar/O_2_ plasma, the value of the contact angle increased from 69.6 ± 2.7° to 81.4 ± 5.7°. On the other hand, after the MD process, a decrease in contact angle was generally found. This is consistent with the fact that during membrane storage, soluble compounds could be formed on their surface, which, in turn, during the MD process are dissolved and finally could lead to increasing of the membranes’ hydrophilicity. 

**Figure 12:**
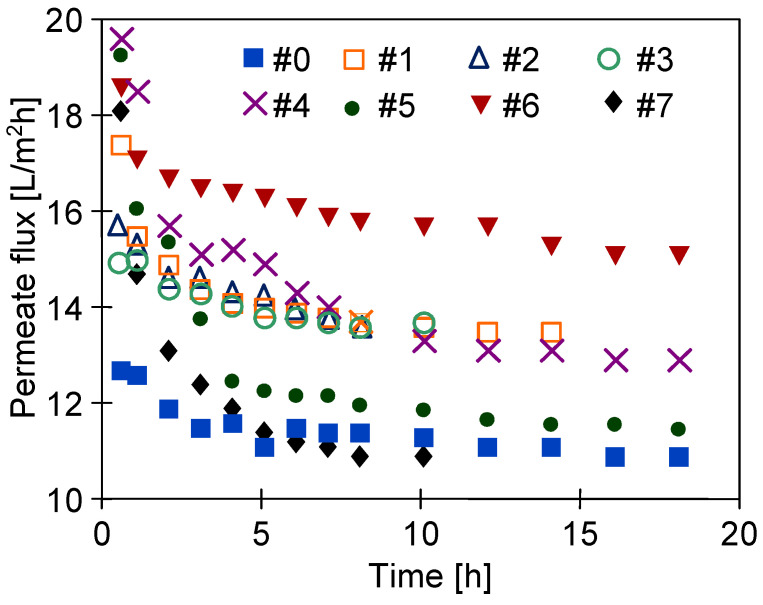
Changes in permeate flux during water desalination by the MD process performed after 4 years of membrane storage.

Figure 12 shows the changes in permeate flux during water desalination by the MD process performed after 4 years of membrane storage. It is worth noting that, similar to the membranes tested 4–5 weeks after the plasma treatment process (Figure 9), a significant decrease in the permeate flux value was observed during the first 2–5 h of the MD process run. This decrease was not observed when the modules were reused after drying (Figure 10). This indicates that plasma-modified membranes stabilized during the first few hours of operation.

**Figure 13:**
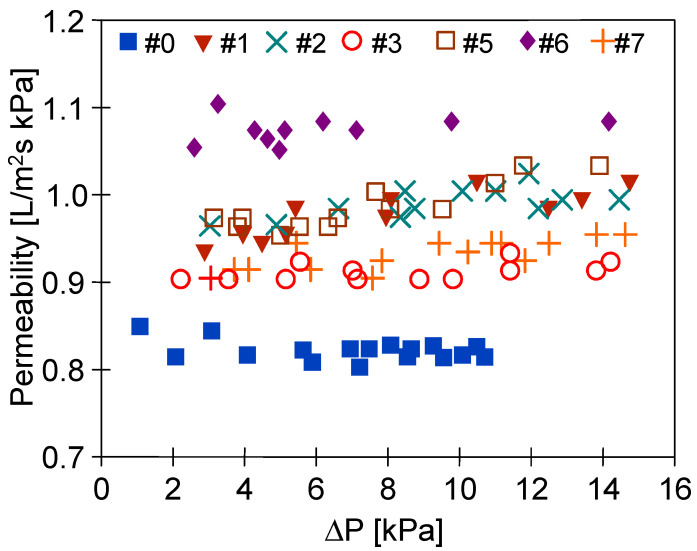
Permeability of membranes treated by plasma (non-used).

**Figure 14:**
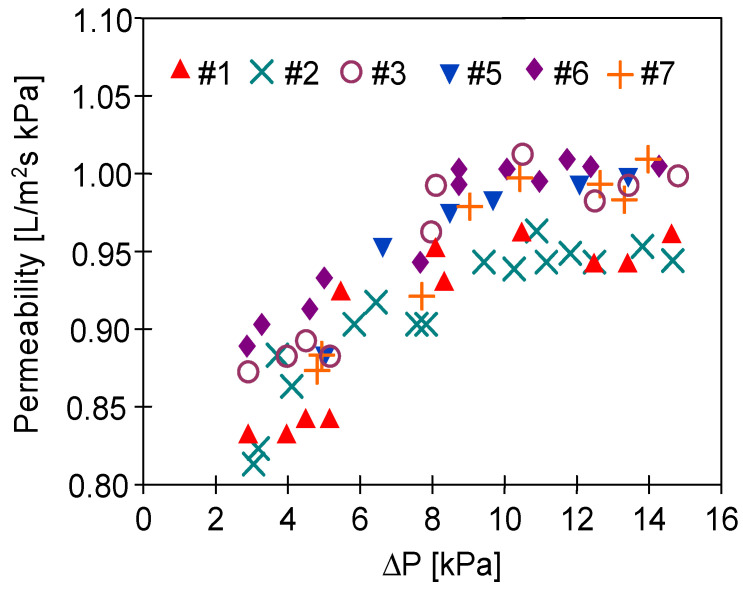
Permeability of membranes treated by plasma (after the MD process).

**Figure 15:**
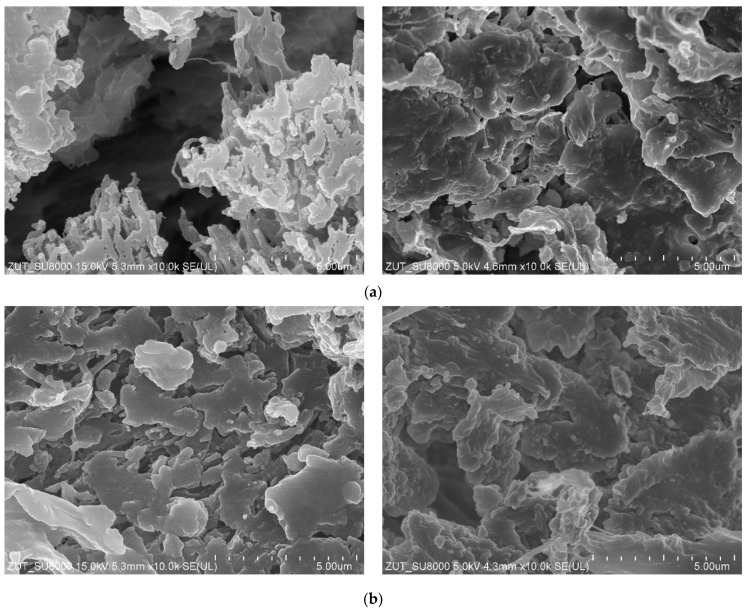

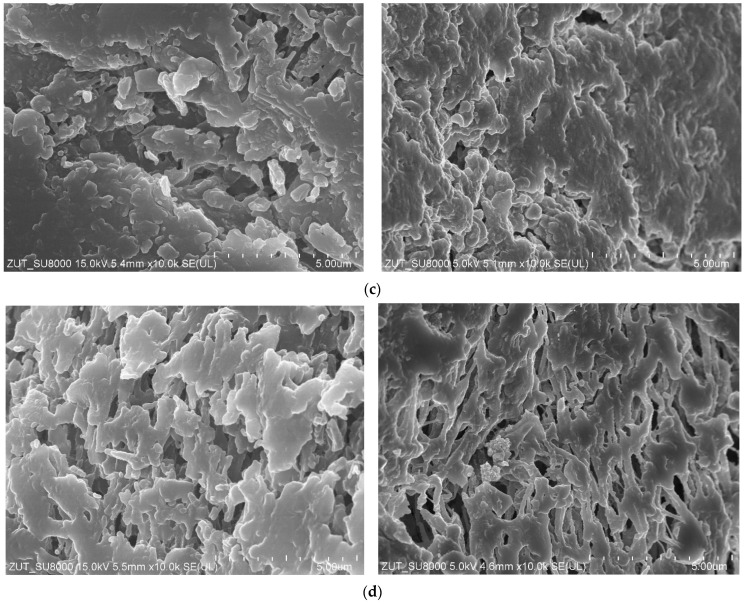
SEM images of membrane samples before (the **left** side) and after the MD process (the **right** side). Membranes: (**a**) #1, (**b**) #2, (**c**) #5, (**d**) #6.

After the MD processes were completed, the membranes were rinsed thoroughly with distilled water and dried naturally in air. Subsequently, the investigation of the impact of the MD process runs on the membranes’ air permeability has been carried out. The results presented in Figure 13 and Figure 14 confirmed that conducting the MD processes led to a decrease in the membranes’ permeability. This can be explained by the fact that the feed temperature was high (353 K), which could lead to some changes in morphology and densification of the membrane structure. Indeed, SEM studies (Figure 15) confirmed that the structure of the membrane surface was more compact after the MD process.

The gas permeability tests were carried out by feeding the gas inside the capillaries, gradually increasing the amount of air forced (Figure 14—increasing value of ΔP). The permeability increased with the increase of the ΔP until the value of 1 L/m^2^ s kPa for the pressure difference above 8 kPa had been achieved. This result indicates that the gas flowing through the wall could cause stretching of the pores on the outer surface of the capillaries (Figure 15) and this effect should increase with increasing ΔP. This observation indicates a certain instability of the plasma-modified layer and no degradation of the polymer since PP had retained its flexibility.

**Figure 16:**
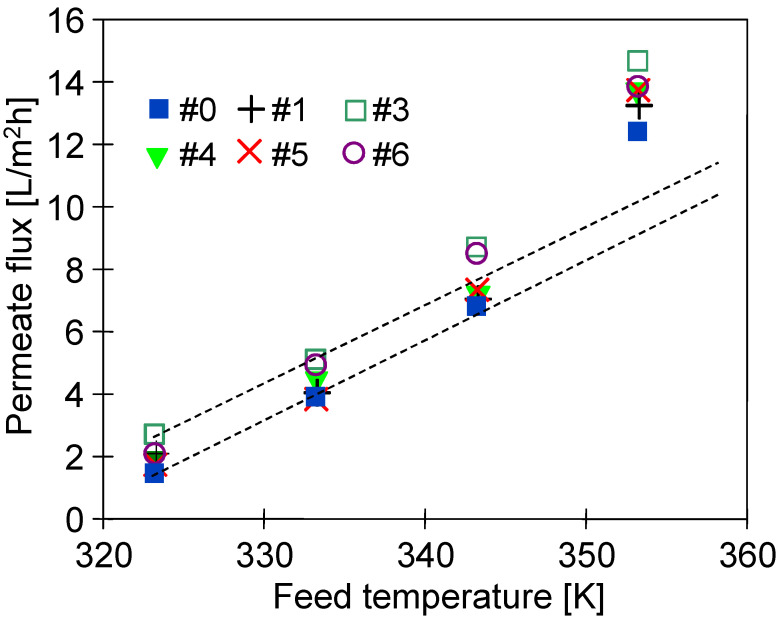
Impact of the feed temperature on the permeate flux. Feed: NaCl solution (3 g/L). Five years after plasma treatment.

After completion of the abovementioned tests, the dried modules as well as unused membranes were stored in the air atmosphere. One year later (corresponding to 5 years from the plasma modification), the investigation of the modules’ performance was performed. It has been found that the used membranes were still efficient. Indeed, the exponential dependence of the permeate flux on temperature typical of the MD process has been noted (Figure 16). Each module was tested for 8 h and the data presented in the figure correspond to the arithmetic mean of two one-hour measurements. NaCl solution (3 g/L) was used as a feed and the obtained distillate was characterized by conductivity below 5 µS/cm. Such a low value indicated that the membranes possessed robust wetting resistance. It is worth noting that for the feed temperature equal to 353 K, the obtained values of the permeate flux were similar to those noted after stabilization in the initial stage of the studies (Figure 9). This result evidenced that the observed changes in the surface morphology (Figure 15) occurred in the initial period of the MD process, after which the membrane structure was stable.

### 3.5. Desalination of Oily Water

As established earlier [26], the modification of membranes with He plasma allowed one to obtain membrane properties limiting the intensity of the oil fouling phenomenon. It has been indicated that these properties resulted from the formation of hydrophilic groups containing C=O on the membrane surface. Therefore, in the next stage of the presented study, the 5-year modules were used to study the desalination of NaCl solution (4 g/L) contaminated by oil emulsion (100 ± 20 mg/L). For this purpose, a set of experiments was carried out.

**Figure 17:**
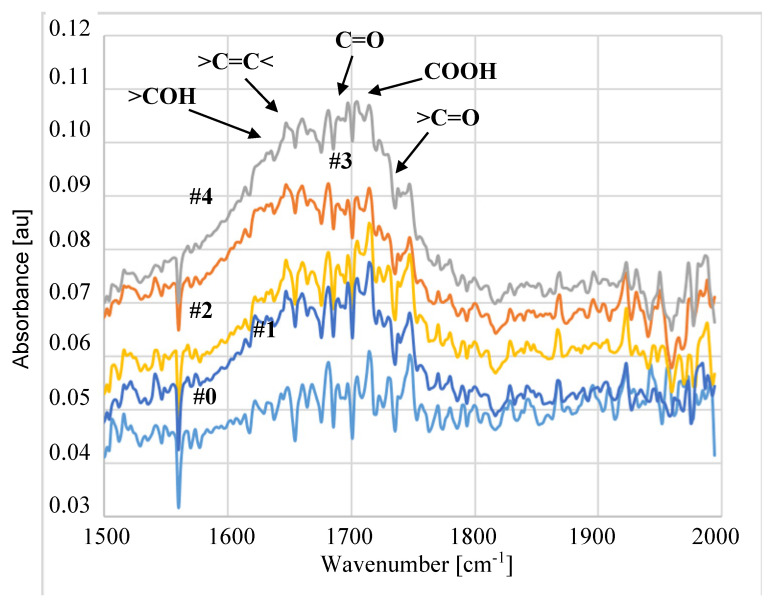
Results of Attenuated Total Reflection-Fourier Transform Infrared Spectroscopy (ATR-FTIR) analyses. Membrane treated by Ar plasma.

**Figure 18:**
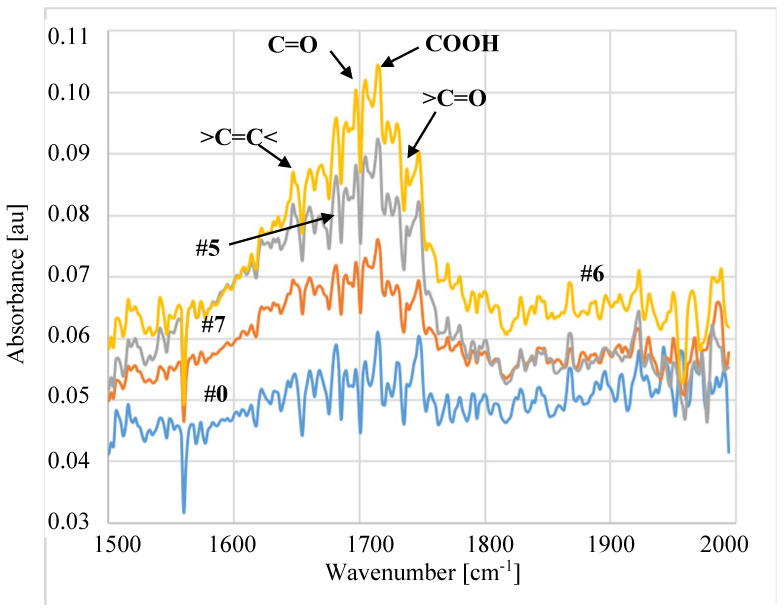
Results of ATR-FTIR analyses. Membrane treated by Ar/O_2_ plasma.

The performed ATR-FTIR studies (Figure 17 and Figure 18) showed that despite 5 years of membrane storage, on the surface of modified membranes, the plasma carbonyl groups were still present in the detected hydrophilic groups, such as COOH or >C=O. The results of ATR-FTIR analysis performed after 5 years of the membrane storage were compared with the results obtained by using the same apparatus after 3 and 4 years. Using the software, diffraction patterns from individual years were superimposed and no significant differences were found. Indeed, the positions of the individual peaks were consistent and their intensity was similar. This result clearly shows that in the following years, despite the membranes being stored in the air, their surface did not degrade. FTIR analysis of the tested membranes was also performed one week after plasma exposure. The performed studies were performed with a different apparatus, which makes their direct comparison difficult, but in each case the plasma-modified samples showed the presence of hydrophilic groups shown in Figure 17 and Figure 18. Compared to the membrane #0, peak intensity for membranes #1–#7 in the range of 1500–1800 cm^−1^ was 3–4 higher, similar to the membranes after 5 years of storage. This result confirms the findings of the initial studies (Figure 5) that the application of higher plasma power (205 W) allows one to obtain stable changes on the membrane surface.

The FTIR tests showed that, regardless of the gas plasma composition (pure Ar or Ar/O_2_), the formation of similar types of hydrophilic groups was detected on the membrane surface (Figure 17 and Figure 18). This observation confirms the results obtained in other studies [37,39] showing that during the contact of membranes with air, free radicals induced by the action of Ar plasma cause the formation of oxygen-containing groups. In the tested samples, the following groups were detected, e.g., >COH (1630 cm^−1^ [50]), C=O (1713 cm^−1^ [50,51]), COOH (1760 cm^−1^ [44]), >C=O (1735 cm^−1^ [34,51]). 

**Figure 19:**
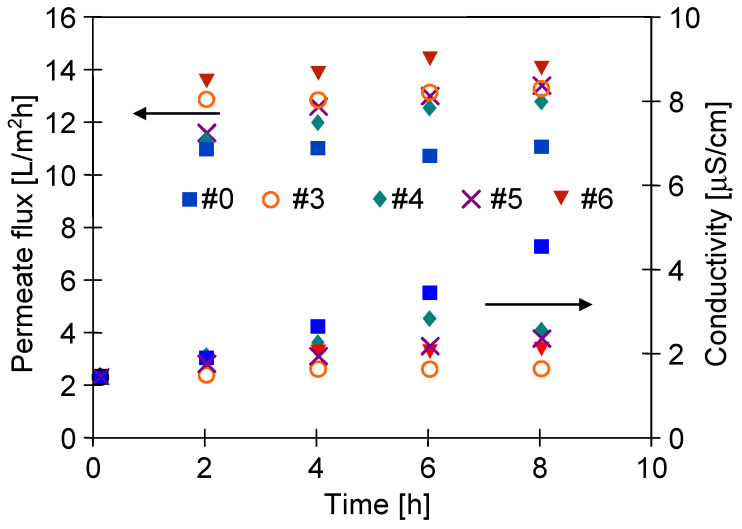
Changes in the permeate flux and distillate conductivity during MD process with the #0 membrane and plasma-treated membranes (#3–#6). Five years after plasma treatment.

The presence of hydrophilic groups confirmed by FTIR tests should limit the intensity of the oil fouling phenomenon during the MD process of salt solution. The adsorption of oil on the membrane surface reduced the feed access to the pores, hence, in this case, a decrease in the process performance is observed. However, the obtained results showed that the module performance was stable (Figure 19). Fouling also leads to wetting of the pores, which results in an increase in the distillate conductivity. It has to be pointed out that in the process performed using the membranes treated by plasma, the conductivity did not increase. Moreover, it has been found that it was similar to that noted for distilled water. Thus, the conducted experiments showed that the used membranes had high wetting resistance. It should be pointed out that the reported value of distilled conductivity for the #0 membrane was twice as high as that for the plasma-modified membranes. This observation clearly indicates that the modified membranes are characterized by greater resistance to fouling.

**Figure 20:**
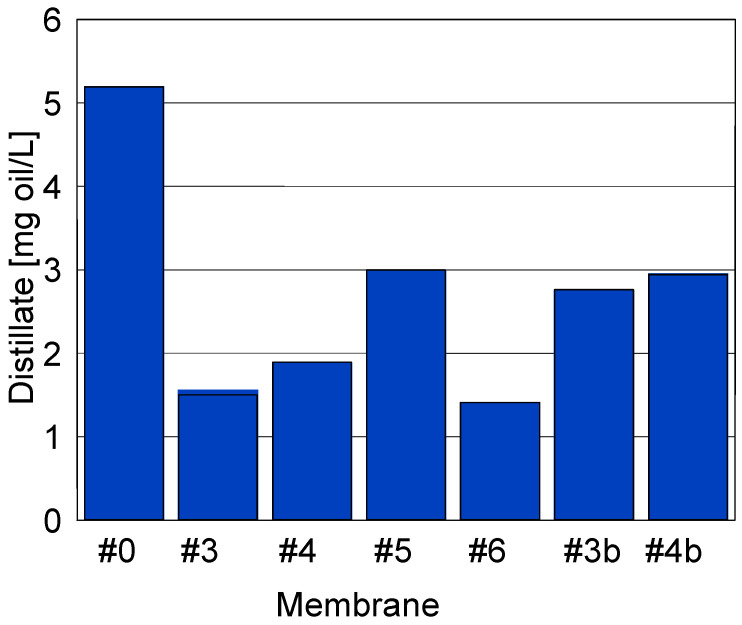
Oil concentration in the obtained distillate.

**Figure 21:**
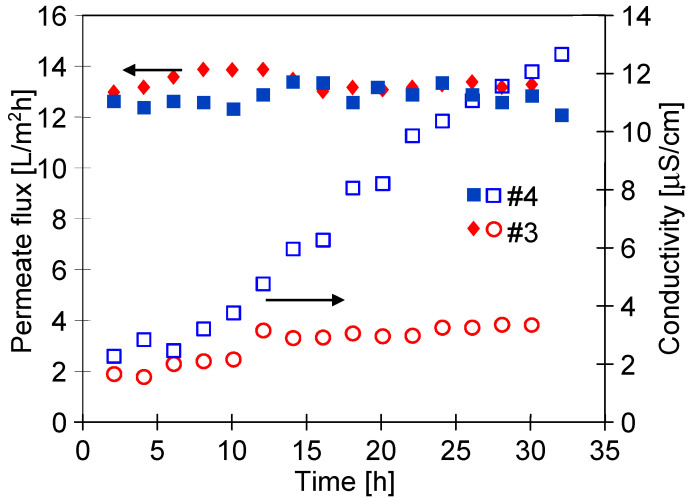
Changes in the permeate flux and conductivity during the long-MD of feed contaminated by oil. Feed: NaCl solution (4 g/L) with oil emulsion (100 ± 20 mg/L).

Moreover, the investigation of the oil concentration in the distillate (Figure 20) has indicated that plasma-treated membranes ensure obtaining permeate of a higher quality that the #0 membrane. The oil content increased slightly (#3b and #4b—Figure 20) during the 3-fold-longer MD tests (Figure 21). For completeness, it should be noted that for these experiments, the membranes showed a stable permeate flux value. For the #4 membrane, a greater increase in the conductivity of the distillate was noted, which indicates that this membrane was slightly more susceptible to wetting. It was due to this fact that membrane #4 had a smaller contact angle than that noted for membrane #3 (Table 3). As a result of the more hydrophilic surface, the salt retention of membrane #4 was also lower and the conductivity of the distillate increased to 13 μS/cm (Figure 21). 

## 4. Conclusions

This paper presents a successful method of Ar/O_2_ plasma treatment applied for a modification of polypropylene membrane surface. As a result of the etching process, the membrane surface changed significantly to a depth of less than 1 µm and surface porosity increased significantly. This facilitated mass transport and led to increasing of the permeate flux by more than 15%. Despite significant surface changes on the membrane surface, the performed long-term studies have confirmed that polypropylene membranes can be successfully used in the MD process for water desalination. The structure of plasma-modified surface stabilized during the first few hours of the MD process, and then remained stable in the following years. Consequently, after 5 years, the membranes were characterized by good stability and high wetting resistance. Contamination of the feed with petroleum compounds causes their adsorption on the membrane surface, which leads to lowering of distillate quality. In the present work, the intensity of this phenomenon was limited by using the polypropylene membranes treated by plasma. Indeed, the use of Ar and Ar/O_2_ gas plasma allowed for the formation of hydrophilic groups on the membrane surface, which limited the adsorption of hydrophobic pollutants. The new properties of PP membranes obtained as a result of the plasma treatment were stable over several years of experiments. Particularly good properties were obtained under higher plasma power (205 W) and flow gas of 90 mL/min. Finally, it should be emphasized that the performed plasma treatment process did not contribute to the degradation of the polymer. Indeed, the 5-year-old modules had properties similar to those noted immediately after their plasma modification. 

## Figures and Tables

**Table 1:** Characteristics of the PP membranes used in the experiments (manufacturer’s data).

Inner Diameter (mm)	Wall Thickness (mm)	Porosity (%)	Average Pore Diameters (µm)
1.8	0.4	72	0.2

**Table 2:** Influence of plasma parameters on the water contact angle and membrane tortuosity.

Membrane	Power (W)	Bias (V)	Time (min)	Plasma	Water Contact Angle (°)	R_A_ (nm)
#0	-	-	-	-	102.1	150 ± 29
#1	205	180	5	Ar	65.1 ± 3.7	286 ± 71
#2	205	270	3	Ar	57.8 ± 2.5	236 ± 63
#3	205	272	1	Ar	61.9	275 ± 68
#4	101	100	5	Ar	62.2 ± 2.9	200 ± 54
#5	205	274	5	Ar/O_2_	71.6	182 ± 80
#6	205	215	5	Ar/O_2_	55.6 ± 2.7	188 ± 55
#7	101	139	5	Ar/O_2_	62.4 ± 2.1	197 ± 20

**Table 3:** Changes of membranes’ water contact angle.

Membrane	Plasma	After 1 Day	After 4 Years	After MD
#0	-	103.9 ± 1.6°	104.7 ± 1.9°	90.1 ± 4.9°
#1	Ar	64.8 ± 2.9°	98.4 ± 2.1°	88.4 ± 3.7°
#2	Ar	62.8 ± 3.5°	106.2 ± 3.4°	100.1 ± 2.8°
#3	Ar	61.9 ± 1.4°	95.8 ± 1.6°	78.5 ± 1.1°
#4	Ar	62.1 ± 2.9°	88.7 ± 2.8°	65.8 ± 1.4°
#5	Ar/O_2_	71.6 ± 1.5°	92.9 ± 2.8°	85.8 ± 3.1°
#6	Ar/O_2_	69.6 ± 2.7°	81.4 ± 5.7°	72.4 ± 2.6°
#7	Ar/O_2_	62.4 ± 2.1°	106.1 ± 1.6°	82.7 ± 5.7°

## Data Availability

The data presented in this study are available on request from the corresponding author. The data are not publicly available due to the institutional repository being under construction.
